# Expected impacts of climate change threaten the anuran diversity in the Brazilian hotspots

**DOI:** 10.1002/ece3.4357

**Published:** 2018-07-13

**Authors:** Tiago S. Vasconcelos, Bruno T. M. do Nascimento, Vitor H. M. Prado

**Affiliations:** ^1^ Departamento de Ciências Biológicas Universidade Estadual Paulista Bauru Brazil; ^2^ Programa de Pós‐Graduação em Ciências Biológicas (Zoologia) Universidade Estadual Paulista Botucatu Brazil; ^3^ Campus Anápolis de Ciências Exatas e Tecnológicas Henrique Santillo Universidade Estadual de Goiás Anápolis Brazil

**Keywords:** alpha diversity, amphibia, Atlantic Forest, beta diversity, Cerrado, macroecology

## Abstract

We performed Ecological Niche Models (ENMs) to generate climatically suitable areas for anurans in the Brazilian hotspots, the Atlantic Forest (AF), and Cerrado (CER), considering the baseline and future climate change scenarios, to evaluate the differences in the alpha and beta diversity metrics across time. We surveyed anuran occurrence records and generated ENMs for 350 and 155 species in the AF and CER. The final predictive maps for the baseline, 2050, and 2070 climate scenarios, based on an ensemble approach, were used to estimate the alpha (local species richness) and beta diversity metrics (local contribution to beta diversity index and its decomposition into replacement and nestedness components) in each ~50 × 50 km grid cell of the hotspots. Climate change is not expected to drastically change the distribution of the anuran richness gradients, but to negatively impact their whole extensions (i.e., cause species losses throughout the hotspots), except the northeastern CER that is expected to gain in species richness. Areas having high beta diversity are expected to decrease in northeastern CER, whereas an increase is expected in southeastern/southwestern CER under climate change. High beta diversity areas are expected to remain in the same AF locations as the prediction of the baseline climate, but the predominance of species loss under climate change is expected to increase the nestedness component in the hotspot. These results suggest that the lack of similar climatically suitable areas for most species will be the main challenge that species will face in the future. Finally, the application of the present framework to a wide range of taxa is an important step for the conservation of threatened biomes.

## 1 INTRODUCTION

Current global climate is approximately 0.85°C warmer than 100 years ago and has a projected increase of 1.5–2°C in the mean temperature by 2100 (IPCC, 2014; Pecl et al., 2017). The resulting changes in the temperature and precipitation regimes have already led species: (a) to alter their physiology to tolerate warmer or drier conditions; (b) to change some of their crucial life cycle events to those period of favorable climate (e.g., the timing of breeding or migration); and (c) to shift their distributional ranges in order to track their usual and/or appropriate conditions in the space (Araújo & Peterson, 2012; Bellard, Bertelsmeier, Leadley, Thuiller, & Courchamp, 2012; Pecl et al., 2017). More worrying yet, some species that could not respond to the climate change effects by the three above‐mentioned alternatives have been reported extinct due to climate change direct effects (Pecl et al., 2017 and references therein). Therefore, the community impacts of shifting species are recognized as one of the primary drivers of biodiversity loss (Sala et al., 2000), although biodiversity gains generated by the redistribution and establishment of new native species also impact the structure of biological communities (Primack et al., 2018). The assessment of these climate change impacts on biodiversity mostly depends on extensive historical information of species distributions, which is unavailable or imprecise for most species in the biodiverse tropics. Therefore, the use of Ecological Niche Models (ENM; or Species Distribution Models: SDM; Araújo & Peterson, 2012) has become a widely used tool to anticipate the climate change effects on species distribution of a wide range of taxa and also to generate conservation strategies based on a dynamic changing climate in different regions of the world (Araújo, Alagador, Cabeza, Nogués‐Bravo, & Thuiller, 2011; Aryal et al., 2016; Cuevas‐Yáñez, Rivas, Muñoz, & Córdoba‐Aguilar, 2015; Fois, Cuena‐Lombraña, Fenu, Cogoni, & Bacchetta, 2016; Lemes & Loyola, 2013).

Ecological Niche Models generally characterize the ecological niche of a species usually considering its climatic preferences based on known occurrence records (Elith & Burgman, 2002). Further, the species climatic niche is projected on a bioclimatic envelope of interest, which can be a different geographic region (Giovanelli, Haddad, & Alexandrino, 2008) or different climate scenarios of the same geographic extent (Vasconcelos & Nascimento, 2016). Even whether these ENMs present commission errors (i.e., the model predicts species presence where there is no occurrence record), these errors may be predictions of interest if the intuit is to look for potential or abiotically suitable areas of a species in future climate change scenarios (Araújo & Peterson, 2012). An alternative to this correlative approach is the use of mechanistic niche models. The mechanistic approach considers some taxon‐specific parameters that provide information on population attributes, physiological limits, and/or biotic interactions for a more precise distribution model (e.g., Pacifici et al., 2015). Nonetheless, mechanistic approaches often focus on a single species with conservation relevance because data on population ecology and/or organisms’ physiology are costly and time‐consuming, which thus make the use of correlative niche models appropriate when the only data available are those on species’ occurrence (Pacifici et al., 2015).

Although the use of ENMs to assess the impacts of climate change on the species distribution has been predominantly performed on a species‐specific basis (e.g., Martins, Silva, de Marco, & Melo, 2015; Nabout, Oliveira, Magalhães, Terribile, & de Almeida, 2011; Vasconcelos, 2014; Vasconcelos & Nascimento, 2016), there is an increasing use of ENMs to generate richness estimates from each species model of a given taxa when occurrence records are available for a high number of species (e.g., Brown & Yoder, 2015; García‐Roselló et al., 2014; Vasconcelos, Rodríguez, & Hawkins, 2012). In such cases, not only the species individual response is evaluated, but different community ecology metrics can be assessed regarding the influence of different climate change scenarios. The most common community ecology metric used is the alpha diversity (i.e., the richness values of the sampling units considered) (Jones & Cheung, 2015; Loyola et al., 2013). A less explored metric, which evaluates some different aspects of the community structure, is the application of the beta diversity index (e.g., Molinos et al., 2015). In summary, beta diversity is the variation in species composition among sites, which can be decomposed into replacement and nestedness components (see review in Legendre, 2014). Considering the different expected effects of climate change on species distributions, a predominance of species range expansions would lead to a decrease in the beta diversity of the whole community due to a homogeneization of the species composition across a region. Then, an application could be the selection of fewer areas in the future to represent all species in the context of conservation biogeography (Legendre, 2014; Socolar, Gilroy, Kunin, & Edwards, 2016). Among a wide range of beta diversity indices, Legendre and De Cáceres (2013) and Legendre (2014) have reviewed and proposed a new index that evaluates the *local contribution to beta diversity* (LCBD hereafter), which is an indicator of ecological uniqueness of the sites for beta diversity. Moreover, the LCBD index can be properly estimated and mapped for macroecological purposes. However, there is little incorporation of this index when evaluating the beta diversity structure of communities modeled under different climate change scenarios.

**Figure 1:**
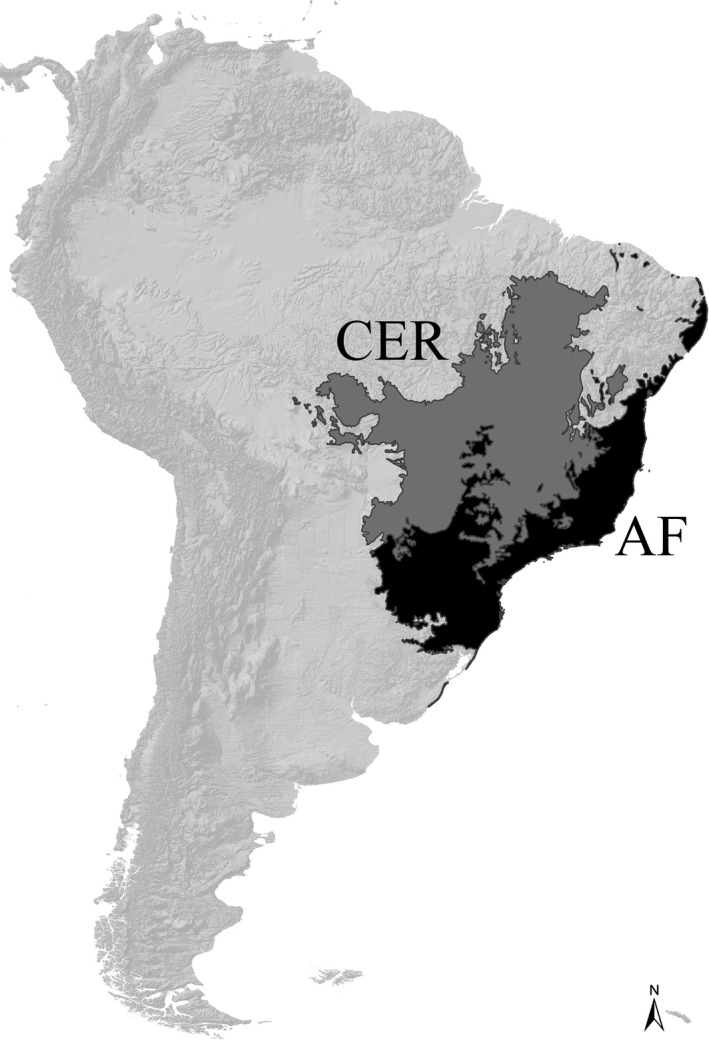
Extent of occurrence of the Atlantic Forest and Cerrado hotspots of biodiversity conservation in South America. Source: originally downloaded from the Conservation International portal (http://www.conservation.org/where/priority_areas/hotspots/Documents/CI_Biodiversity-Hotspots_2011_ArcView-Shapefile-and-Metadata.zip), currently available at http://www.cepf.net/SiteAssets/hotspots_2016_1.zip

In this study, we evaluate the alpha and beta diversity metrics of anuran amphibians in the Brazilian hotspots of biodiversity conservation (Figure 1): the Atlantic Forest (AF) and Cerrado (CER) (sensu Mittermeier et al., 2004). Anurans are highly diverse in the AF, with approximately 550 species, of which ~80% are endemic to this hotspot (Haddad et al., 2013). The main determinants of this high diversity have been attributed to the high humidity levels and number of aquatic micro‐habitats that may have led to the diversification of different reproductive modes, mainly in the moist forests, the rough topography that may have promoted speciation of micro‐endemic species, and the climatic stability of some areas since the Pleistocene glaciations (da Silva, Almeida‐Neto, Prado, Haddad, & Rossa‐Feres, 2012; Haddad & Prado, 2005; Thomé et al., 2010; Vasconcelos, Santos, Haddad, & Rossa‐Feres, 2010). With a predominance of open vegetation formations, less humidity, and a markedly seasonal climate, anurans in the CER are less diverse than in the AF. There are 209 anuran species recorded in the CER, yet high levels of endemism rates are also recorded in this area (approximately 51.7% are endemic species) (Valdujo, Silvano, Colli, & Martins, 2012).

We evaluate here how the effects of climate change are presumed to impact the species richness gradients and the beta diversity structure of anurans in the AF and CER Brazilian hotspots. Specifically, we make use of ENMs to generate the climatically suitable area of the species, considering the baseline and future climate change scenarios, in order to answer the following questions: (a) How is climate change expected to change the anuran richness gradients in the AF and CER? (b) Where, in the geographical space, are the areas located with predicted losses and gains in species richness? (c) How is climate change expected to change the location of areas having unique species composition (i.e., high beta diversity values)? and (d) How is climate change expected to change the structure of anuran beta diversity (i.e., the relative importance of nestedness and turnover components) in the AF and CER?

## 2 MATERIALS AND METHODS

### 2.1 Species data

Based on the work of Haddad et al. (2013) and Valdujo et al. (2012), there are 529 and 209 anurans occurring within the AF and CER, respectively. First, we surveyed the geographic coordinates of these species from two open‐access digital databases that provide occurrence data from biological surveys and museum collections (the Global Biodiversity Information Facility, GBIF: http://www.gbif.org; the SpeciesLink project: http://splink.cria.org.br). Due to the geographic bias on occurrence records from these online databases (e.g., Beck, Böller, Erhardt, & Schwanghart, 2014; Vasconcelos & Nascimento, 2014), we also made use of the following two sources to collect thorough point occurrence records of each species: a) museum and scientific collections, mainly those not hosted by GBIF and SpeciesLink and/or representative of different regions within the AF and CER: *Museu Nacional/Universidade Federal do Rio de Janeiro* (Rio de Janeiro, RJ, Brazil), *Museu de Zoologia da Universidade Federal da Bahia* (Salvador, BA, Brazil), *Coleção Herpetológica da Universidade Federal de Santa Maria* (Santa Maria, RS, Brazil), and the American Museum of Natural History (New York, NY, USA); and b) literature records focusing on journals prioritizing publications on species list and/or geographic distribution updates. Occurrence records of those species also occurring outside the AF and CER borders were considered in the dataset. All records were carefully examined, species by species, for probable errors. Therefore, we removed duplicate and/or imprecise species records, such as those occurrences outside the geographic species ranges according to Frost (2017) and/or to the pertinent literature of a given species. Records from introduced species were not considered (e.g., *Lithobates catesbeianus* and *Xenopus laevis*). The amphibian nomenclature followed Frost (2017). This survey resulted in a total of 512 anuran species in the AF (with a total of 18,799 occurrence points) and 197 anuran species in the CER (with a total of 16,387 occurrence points) (Supporting Information Tables S1 and S2). After removing those species with fewer than five occurrence records due to modeling limitations (e.g., Hernandez, Graham, Master, & Albert, 2006; Ochoa‐Ochoa, Urbina‐Cardona, Vázquez, Flores‐Villela, & Bezaury‐Creel, 2009; Vasconcelos et al., 2012), we were able to generate the ENMs for 350 anuran species in the AF and 155 species in the CER.

### 2.2 Ecological niche models

We used a continental (South America) calibration area for the ENMs, which means that the climatic niche of each species was projected in the South American bioclimatic envelope. In the present study, the patterns of species geographic distribution vary within a continuum from narrowly to widely ranged. Considering that different calibration areas generate different range size predictions (Giovanelli, de Siqueira, Haddad, & Alexandrino, 2010), performing the ENMs with different calibration areas for each species would become logistically complex and difficult to interpret (Vasconcelos et al., 2012). Then, in order to perform a standardized procedure than to a species‐by‐species treatment regarding the climate variable selection, we considered a single (continental) calibration area for the modeling procedure. From the 19 climatic variables available in the WorldClim portal at a spatial resolution of ~10 km (Hijmans, Cameron, Parra, Jones, & Jarvis, 2005: http://www.worldclim.org/version1), we selected nine variables that are neither collinear nor redundant variables as the baseline climate in the modeling process considering the calibration area: mean diurnal range, isothermality, mean temperature of wettest quarter, mean temperature of driest quarter, precipitation of wettest month, precipitation of driest month, precipitation seasonality, precipitation of warmest quarter, and precipitation of coldest quarter. These variables were selected based on their variance inflation factors (VIFs). We calculated their VIFs and excluded the variables with highest VIF until all variables presented VIF lower than 10.0, thus representing no collinearity problems for model building (Naimi & Araújo, 2016). The same variables, at the same spatial resolution and also available at the WorldClim portal (http://www.worldclim.org/cmip5_5m), were considered to project the baseline climatic niches into future climate change scenarios (2050 and 2070). For each future time slice (2050 and 2070), we used two representative concentration pathways (RCPs) of two CO_2_ emission scenarios (RCP2.6 and RCP8.5) and from three different atmosphere–ocean global circulation models (AOGCMs) of the Intergovernmental Panel on Climate Change (IPCC Fifth Assessment Report): the Community Climate System Model (CCSM4), the New Global Climate Model of the Meteorological Research Institute (MRI‐CGCM3), and the Institute Pierre Simon Laplace (IPSL‐CM5A‐LR).

We generated the ENMs using four modeling algorithms: generalized linear models (GLM), boosted regression trees (BRT), random forests (RF), and support vector machines (SVM). These are statistical (GLM) and machine‐learning methods (BRT, RF, and SVM) known to produce reasonably reliable results, so detailed descriptions and usefulness of these algorithms can be found elsewhere (e.g., Araújo et al., 2011; Elith et al., 2006; Naimi & Araújo, 2016; Vasconcelos, Antonelli, & Napoli, 2017). The records were split into 20% for model evaluation (random test percentage) and the remaining 80% used for calibration (training). The models were evaluated by two metrics (see Allouche, Tsoar, & Kadmon, 2006) that are briefly summarized: (a) the area under the curve (AUC) of the receiver operation characteristic (ROC), a threshold‐independent statistic that ranges from 0 (model equivalent to a random prediction) to 1 (perfect model discrimination between presence and absence records); and (b) the true skill statistic (TSS), a threshold‐dependent statistic that ranges from −1 (model equivalent to a random prediction) to 1 (perfect model fit). Here, ENMs with AUC <0.75 or TSS <0.3 were excluded from the ensemble procedure (see ahead) because their predictive powers were similar to random predictions.

To create a more robust final predictive map of each species for each time slice (current, 2050, and 2070), we generated ensemble forecasts to reconcile the inherent uncertainties generated from the use of different algorithms, AOGCMs, and RCPs (Araújo & New, 2007). Regarding the baseline projections, the climatic suitability map of each species considered the mean value per grid cell of the four algorithms, whereas the 2050 and 2070 projections considered the mean value per grid cell of the four algorithms X 3AOGCMs X 2RCPs (Araújo et al., 2011). Further, we generated a binary map for each species based on the mean threshold value among the different algorithm predictions of the sensitivity‐specificity equality approach (Liu, Berry, Dawson, & Pearson, 2005). The dispersal ability of species under climate change, which means assuming species presence in those climatically suitable sites that are not suitable in the baseline predictions, was only considered for those continuous predictions. That is, any continuous climatically suitable area that partially overlaps the baseline prediction has the potential to be colonized by a given species. On the other hand, this assumption is more critical to isolated climatically suitable areas due to the necessity of a given species to disperse throughout a climatically unsuitable area. Then, we kept the main predicted continuous areas of species irrespective if there are new climatically suitable areas compared to the baseline predictions; yet we individually checked each binary prediction to delete unrealistic isolated predictions (i.e., environmentally suitable areas of a given species instead of predicted distributions) more than 400 km away from the main predicted area or any known occurrence record of a species (Vasconcelos et al., 2012). Finally, these binary maps were used to create a presence–absence matrix to determine the alpha and beta diversity metrics for each time slice. All pre‐processing (preparation of species and climate data), processing (model running), and post‐processing (model evaluation and ensemble maps) procedures were performed using the *sdm* package in the R environment (Naimi & Araújo, 2016).

### 2.3 Assessing climate change impacts on alpha and beta diversity

Here, we consider the patterns of alpha diversity (i.e., grids representing local sites) as a representation of local species richness (e.g., Davey, Devictor, Jonzén, Lindström, & Smith, 2013; Molinos et al., 2015). To do so, each predictive binary species map was summed up in a ~50 × 50 km grid system of the AF and CER, a procedure repeated for each time slice. The richness values of each grid cell were compared with the future against current richness predictions by paired *t* tests to evaluate the effects of climate change on the anuran alpha diversity, and were also used to make up ladder plots with the predicted richness grid values across time slices (e.g., Loyola, Lemes, Brum, Provete, & Duarte, 2014). Additional calculation of gains and losses in species richness between future and current predictions, as well as the confection of final maps, were performed using ArcGIS10.1.

The patterns of AF and CER anuran beta diversity for different time slices were considered by the calculation of the LCBD index, as proposed by Legendre and De Cáceres (2013). In summary, the presence/absence anuran matrix, independently performed for different time slices for each hotspot, was submitted to the Jaccard dissimilarity coefficient (Bjac) in order to compute the total variance of the community composition. Then, the LCBD was determined based on the partition of total beta diversity among the sites, obtained from the Jaccard dissimilarity matrix, to obtain the relative contribution of sampling units (i.e., grid cells) to beta diversity. The LCBD values can be mapped and represent comparative indicators of the ecological uniqueness of the sites in terms of community composition. That is, the LCBD values indicate the sites that contribute more (or less) than average to beta diversity; large LCBD values may indicate sites having unusual species combinations and of high conservation values compared to the total local (i.e., grid cell) communities (Legendre & De Cáceres, 2013). We also tested the significance of the LCBD values of each cell by performing permutations in which the species are random and independently distributed of one another among the grid cells (Legendre & De Cáceres, 2013).

In order to explore whether the structure of the anuran beta diversity is expected to change under climate change, we decomposed the Jaccard dissimilarity coefficient into replacement and nestedness components (i.e., the replacement and nestedness index of the Baselga‐family, presence‐absence data, sensu Legendre, 2014). Therefore, we aimed to check whether and which of the beta diversity components (the species turnover among sites or the nested pattern characterized by the species at a site being a strict subset of the species at a richer site) are presumed to vary in future climate change scenarios. Since the replacement (Repl) and nestedness (Nes) indices equal the dissimilarity (D) index, and that the similarity (S) index equals 1 – D, we are following Podani and Schmera (2011) and Podani, Ricotta, and Schmera (2013) and are representing the triplets of values (S, Repl, Nes) in a triangular graph for each time slice and hotspot (see also Legendre, 2014). Each triplet sums to 1 and the central dot is the centroid of the points, so the graphics illustrate the main beta diversity component structuring the anuran community at each time slice. The determination of LCBD indices and decomposition of beta diversity into replacement and nestedness components were performed in R with the scripts provided by Legendre and De Cáceres (2013) and Legendre (2014), whereas the triangular graphs were generated using the ade4 package (Chessel, Dufour, & Thioulouse, 2004).

## 3 RESULTS

### 3.1 Model performances and losses in climatic suitability across time

All four modeling methods had strong predictive power, with mean AUC ± *SD* and mean TSS ± *SD* values of 0.938 ± 0.069 and 0.863 ± 0.139 (BRT), 0.936 ± 0.065 and 0.871 ± 0.129 (GLM), 0.973 ± 0.045 and 0.928 ± 0.107 (RF), and 0.972 ± 0.052 and 0.934 ± 0.112 (SVM), which indicate good model fit for the species predictions. Of the 350 AF anuran species, 8.29% (29 species) are predicted to have no climatically suitable area by 2050 and 2070, and other eight species will have some climatically suitable area by 2050 but not by 2070 (Table 1). Of the 155 anurans in the CER, only 1.93% (three species) are presumed to have no climatically suitable area by 2050 and 2070, and two more species that have some predicted area by 2050 but are presumed to totally lose their climatically suitable areas by 2070 (Table 1).

**Table 1:** Atlantic Forest (AF), and Cerrado (CER) anuran species predicted to have no climatically suitable area by 2050 and/or 2070. Threatened species according to the Brazilian Ministry of the Environment, document 444 of December 17th of 2014, available at http://www.icmbio.gov.br/portal/images/stories/biodiversidade/fauna-brasileira/avaliacao-do-risco/PORTARIA_N%C2%BA_444_DE_17_DE_DEZEMBRO_DE_2014.pdf

Species name	Threatened	Hotspot	Time slice
*Adenomera araucaria*		AF	2050/2070
*Aparasphenodon arapapa*		AF	2050/2070
*Bokermannohyla astartea*		AF	2050/2070
*Bokermannohyla lucianae*		AF	2050/2070
*Cycloramphus semipalmatus*		AF	2050/2070
*Dendrophryniscus berthalutzae*		AF	2050/2070
*Euparkerella tridactyla*		AF	2050/2070
*Holoaden bradei*	X	AF	2050/2070
*Hylodes magalhaesi*		AF	2050/2070
*Hyophryne histrio*		AF	2050/2070
*Hypsiboas joaquini*		AF	2050/2070
*Ischnocnema manezinho*	X	AF	2050/2070
*Leptodactylus viridis*		AF	2050/2070
*Melanophryniscus dorsalis*	X	AF	2050/2070
*Melanophryniscus macroglanulosus*	X	AF	2050/2070
*Melanophryniscus pachyrhinus*		AF	2050/2070
*Melanophryniscus spectabilis*		AF	2050/2070
*Odontophrynus maisuma*		AF	2050/2070
*Phyllodytes edelmoi*		AF	2050/2070
*Physalaemus erythros*		AF	2050/2070
*Physalaemus moreirae*		AF	2050/2070
*Physalaemus obtecus*		AF	2050/2070
*Physalaemus soaresi*	X	AF	2050/2070
*Scinax littoreus*		AF	2050/2070
*Scythrophrys sawayae*		AF	2050/2070
*Sphaenorhynchus bromelicola*		AF	2050/2070
*Sphaenorhynchus orophilus*		AF	2050/2070
*Stereocyclops parkeri*		AF	2050/2070
*Strabomantis aramunha*		AF	2050/2070
*Bokermannohyla nanuzae*		AF/CER	2070
*Cycloramphus juimirim*		AF	2070
*Cycloramphus lutzorum*		AF	2070
*Hypsiboas curupi*	X	AF	2070
*Ischnocnema parva*		AF	2070
*Physalaemus erikae*		AF	2070
*Proceratophrys schirchi*		AF	2070
*Scinax littoralis*		AF	2070
*Bokermannohyla alvarengai*		CER	2050/2070
*Leptodactylus camaquara*		CER	2070
*Scinax pinima*		CER	2050/2070
*Trachycephalus mambaiensis*		CER	2050/2070

### 3.2 Alpha diversity

**Figure 2:**
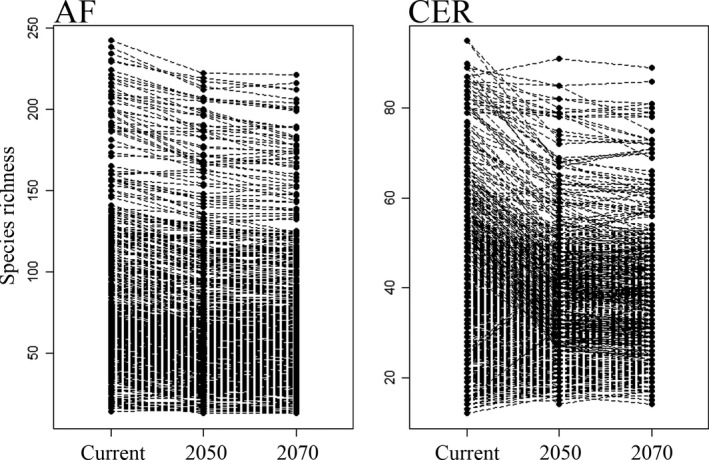
Ladder plots of Atlantic Forest (AF) and Cerrado (CER) anuran richness values per grid cell for the current baseline, 2050, and 2070 climate change scenarios based on the overlap of climatic suitability of species generated by ENM

**Figure 3:**
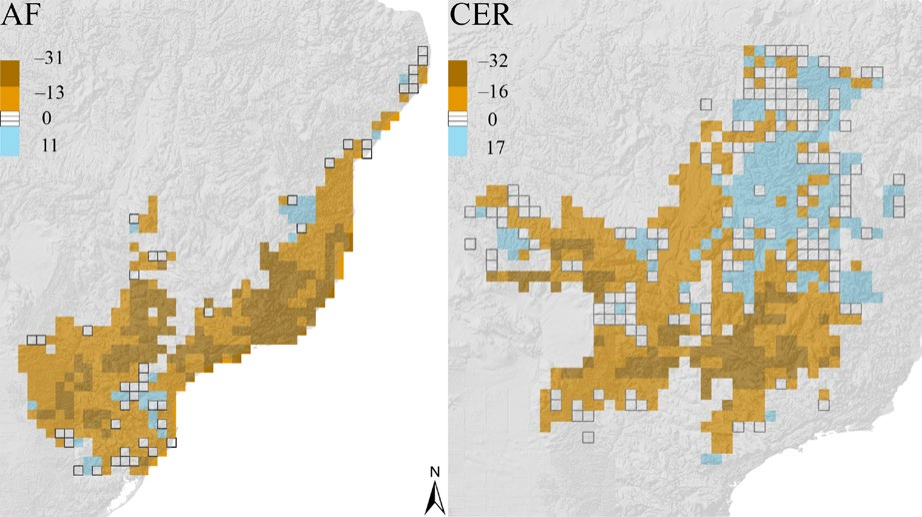
Geographic distribution of gains (blue grids) and losses (brown grids) of the species richness in the Atlantic Forest (AF) and Cerrado (CER) between the baseline climate and 2050 predicted anuran richness based on the overlap of climatic suitability of species generated by ENM

**Figure 4:**
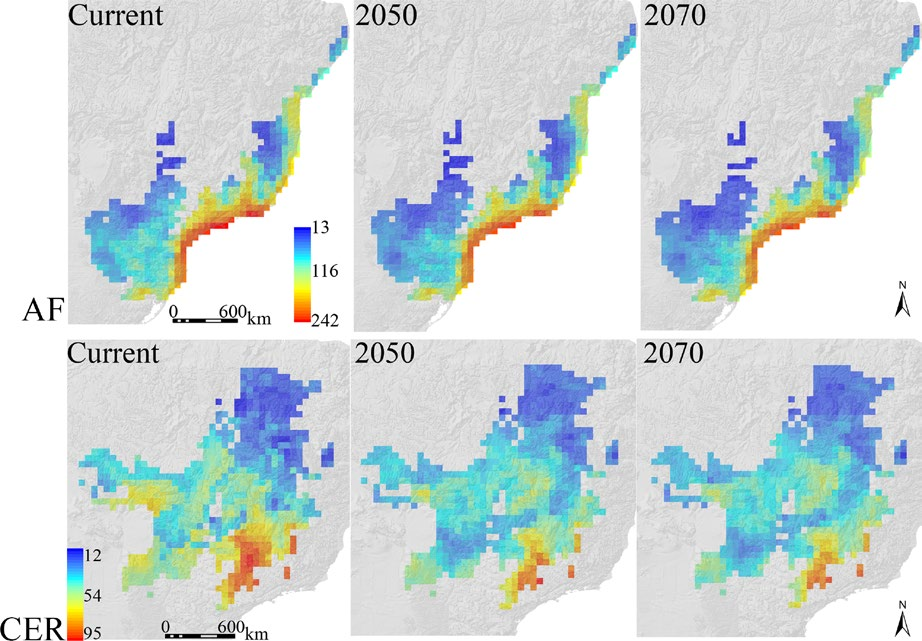
Geographic distribution of the anuran richness gradients in the Atlantic Forest (AF) and Cerrado (CER) for the baseline climate, 2050, and 2070 climate change scenarios based on the overlap of climatic suitability of species generated by ENM

The mean richness per grid is expected to decrease across time in the AF (current‐2050: *t* = −25.45, *p* = 0.000; current‐2070: *t* = −13.76, *p* = 0.000; 2,050–2,070: *t* = −5.489, *p* = 0.000) and in CER (current‐2050: *t* = −12.947, *p* = 0.000; current‐2070: *t* = −13.76, *p* = 0.000; 2,050–2,070: *t* = −5.489, *p* = 0.000) (Figure 2), although gains in species richness can be expected in future scenarios for some specific regions of both hotspots, but mainly in the northeastern region of CER (Figure 3). The maximum richness value reached by a grid cell in the AF is 242 species for the baseline climate, 222 for 2050, and 221 species for the 2070 climate change consensus scenarios (Figure 4). This decreasing tendency is also found for the CER, in which 95 species are predicted in the richest cell for the baseline climate, 91 for 2050, and 89 species for the 2070 climate change consensus scenario (Figure 4). Regarding richness gradients, the climate change scenarios do not change the fact that the higher anuran species richness is found in the coastal rim of the AF, mainly in southeastern Brazil, whereas the lower anuran richness is found in inland places, away from the Atlantic coast (Figure 4). Similarly, anuran richness in the CER is broadly congruent among different climate change scenarios; the higher species richness is found in southeastern areas of the CER and, to a lesser extent, some central and southwestern areas, whereas lower richness is found in the northern and northeastern Cerrado (Figure 4). The major impacts of climate change on the richness gradients in the AF are found in specific inland regions of the southeastern and southern Brazil where anuran richness is expected to decrease by 2050 and 2070 (Figures 3 and 4). Though the decrease in anuran richness is expected to happen in the southeastern, southwestern, and western regions of the CER by 2050 and 2070, most northeastern grids of the CER are expected to gain in species by 2050 (Figures 3 and 4).

### 3.3 Beta diversity

**Figure 5:**
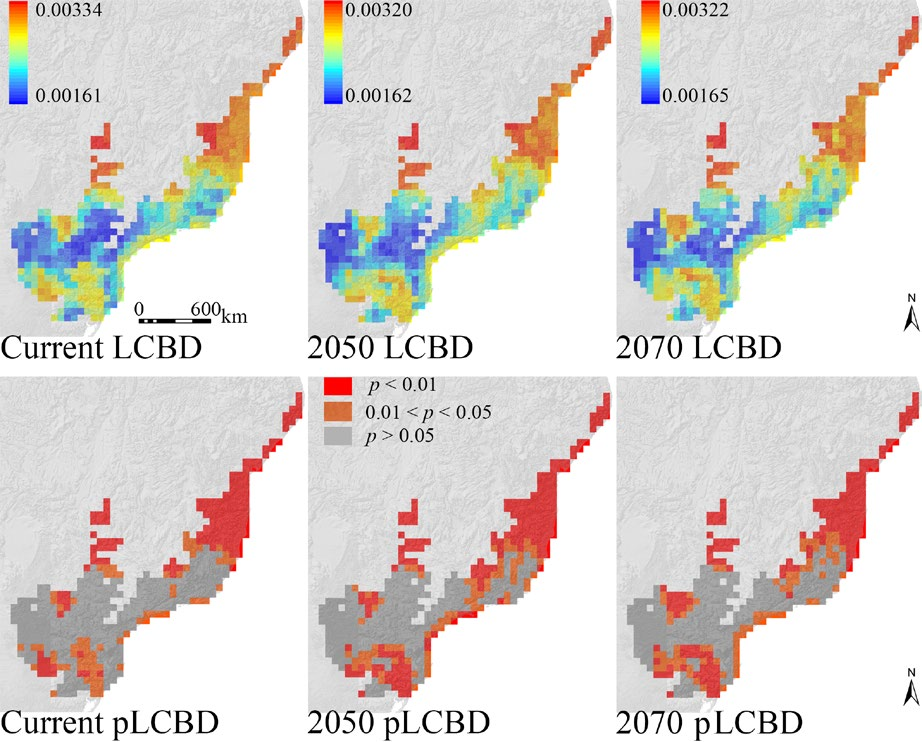
Geographic distribution of the local contributions to beta diversity (LCBD) of the Atlantic Forest anuran assemblages for the current baseline, 2050, and 2070 climate change scenarios based on the overlap of climatic suitability of species generated by ENM. Lower maps highlight significant LCBD values at the 0.05 significance level (pLCBD)

**Figure 6:**
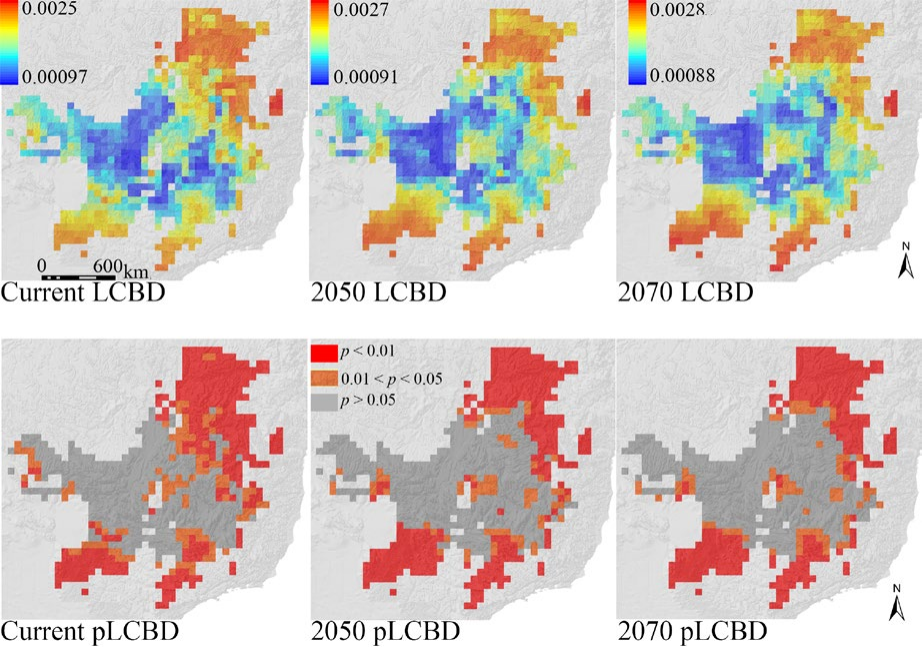
Geographic distribution of the local contributions to beta diversity (LCBD) of the Cerrado anuran assemblages for the current baseline, 2050, and 2070 climate change scenarios based on the overlap of climatic suitability of species generated by ENM. Lower maps highlight significant LCBD values at the 0.05 significance level (pLCBD)

The patterns of beta diversity distribution in the AF depict that unique anuran compositions are found in the whole northern region, in a narrow region in the southeastern coast, in part of the southern region, and in some specific inland regions in transition zones with the CER (Figure 5). This pattern is overall similar among the current, 2050, and 2070 predictions, but the main difference among them is an increase across time in high beta diversity neighboring cells of the mentioned high beta diversity regions (Figure 5). The dissimilarity values of anuran beta diversity in the AF show that the level of changes in the anuran composition across time is quite similar among them (current Bjac = 0.354; 2050 Bjac = 0.355; 2070 Bjac = 0.359). In the CER, all predictions depict high anuran beta diversity in the northern/northeastern, southeastern/southwestern, and fewer regions in the central and western regions of the hotspot (Figure 6). The main difference among the current, 2050, and 2070 predictions is that the climate change scenarios identify a wider southeastern/southwestern area having high anuran beta diversity when compared to current predictions, whereas a smaller area in the northeastern portion of the CER is predicted for the future scenarios (Figure 6). The anuran composition in the CER is presumed to become more homogeneous in future climate change scenarios since the average anuran beta diversity decreases from the current (current Bjac = 0.331) to future predictions (2050 Bjac = 0.303; 2070 Bjac = 0.298).

**Figure 7:**
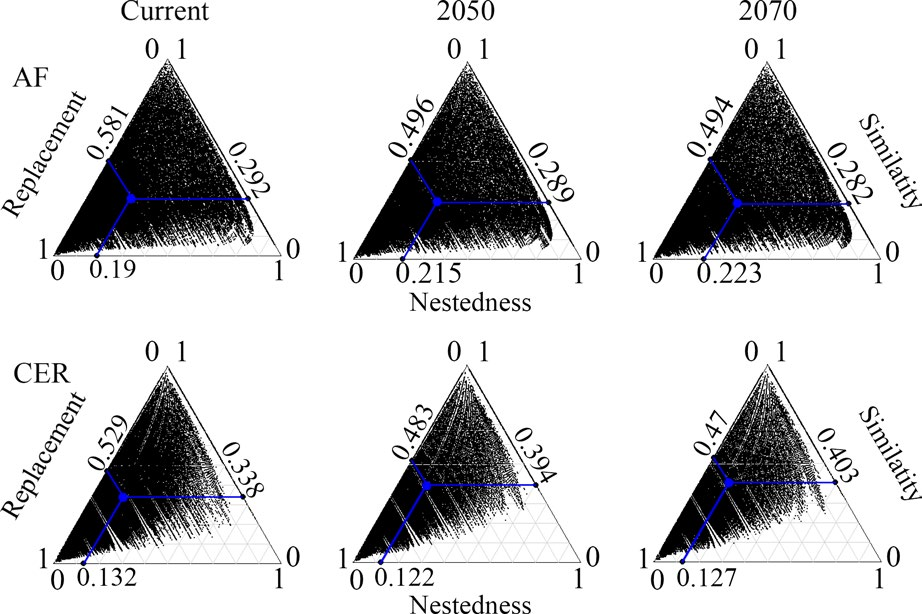
Triangular plots of the relationships among the pairs of grid cells (black dots) for the Atlantic Forest (AF) and Cerrado (CER) anuran assemblages decomposed from the Jaccard dissimilarity coefficient into replacement and nestedness components. Large central blue dots in each graph are the centroids of the respective mean values (blue lines) of the similarity, replacement, and nestedness components

The partitioning of beta diversity into components of spatial turnover and nestedness reveals that the replacement of species among the grids is the main component of the beta diversity for both hotspots and time slices (Figure 7). Though the turnover component remains the main component of the beta diversity of both hotspots in the future, the climate change scenarios are expected to cause different changes in the importance of the beta diversity components of each hotspot. In the AF, although the similarity values of beta diversity tend to remain similar among the current, 2050, and 2070 predictions, as mentioned above; the nestedness component becomes higher in future climate change scenarios than the baseline predictions while the turnover component shows the inverse pattern (i.e., the replacement values decrease across time) (Figure 7). In the CER, either the turnover and nestedness components of beta diversity decrease from baseline to future climate scenarios, with an associate increase in the similarity values of the Bjac in future predictions (Figure 7).

## 4 DISCUSSION

The first expected impact of climate change on the AF and CER anurans is the extinction of 42 species (37 species in the AF and five in the CER) by means of their complete loss of climatically suitable areas by 2050 and 2070. Lemes, Melo, and Loyola (2014) and Loyola et al. (2014) already documented predicted extinctions of some AF anurans by 2050, either in a smaller (Lemes et al., 2014; : nine out of 430 species analyzed – 2.09%) or a higher proportion (Loyola et al., 2014: 52 out of 431 species – ~12%) than that recorded herein (37 out of 350 species: 10.57%). These percentage differences may be accounted for the differences in the study designs and performed modeling techniques, but the important message here is that if these anurans no longer have physiological, morphological, or behavioral adaptations to accommodate to novel climatic conditions, or whether they are unable to change their particular timing of life‐history events to avoid the months with unfavorable climates (Bellard et al., 2012), they are presumed to contract their ranges until extinction due to the fact that they have no climatically suitable area predicted by 2050 and/or 2070. In the present study, these predictions are more worrisome for the fate of some already endangered AF anurans, such as *Holoaden bradei*,* Hypsiboas curupi*,* Ischnocnema manezinho*,* Melanophryniscus cambaraensis*,* M. dorsalis*,* M. macrogranulosus*, and *Physalaemus soaresi* (according to the Brazilian Ministry of the Environment, document 444 of December 17th of 2014, available at http://www.icmbio.gov.br/portal/images/stories/biodiversidade/fauna-brasileira/avaliacao-do-risco/PORTARIA_N%C2%BA_444_DE_17_DE_DEZEMBRO_DE_2014.pdf), all of them predicted to have no climatically suitable area by 2050 and/or 2070. Additionally, since decision‐makers use the distribution criteria to evaluate the risk category of species, the threatened status might be higher in the future than the current list due to the tendency for range contraction of species (Lemes et al., 2014; Loyola et al., 2014; present study). This highlights the importance of ENMs to assess the reliability of conservation status under specific present or future threats that could occur in each locality (Fois et al., 2016).

Overall, the local estimates of species richness are overestimated compared to previous macroecological documentations of anuran richness in the AF (e.g., Vasconcelos, Prado, da Silva, & Haddad, 2014; Villalobos, Dobrovolski, Provete, & Gouveia, 2013) and CER (e.g., Diniz‐Filho et al., 2006, 2008). This is probably related to higher rates of commission errors of ENMs over the richness estimates generated by superimposing individual expert range maps (Vasconcelos et al., 2012 and references therein). Nonetheless, the richness gradients for the baseline climate documented here for both hotspots are broadly congruent with these previous studies (Diniz‐Filho et al., 2006, 2008; Vasconcelos et al., 2014; Villalobos et al., 2013).

Studies projecting species’ distributions under climate change generally predict altered patterns of broad‐scale species richness (e.g., Jones & Cheung, 2015; Luo, Jiang, & Tang, 2015; Molinos et al., 2015) as a response of the poleward latitudinal and/or upward altitudinal range shifts of the species (Araújo, Thuiller, & Pearson, 2006; Chen, Hill, Ohlemüller, Roy, & Thomas, 2011). Therefore, it follows that low latitude regions are expected to lose in species richness, whereas high latitude regions have the opposite predictions, i.e. to gain in species richness (Jones & Cheung, 2015; Molinos et al., 2015). As stated in the last paragraph, the richness gradients for the baseline climate documented here for both hotspots are broadly congruent with previous studies. Nonetheless, the climate change is not expected to cause deep changes in the spatial configuration of anuran richness in both hotspots, a prediction already documented for amphibians in the AF (Loyola et al., 2013). This is somewhat expected due to the small climatic gradient of the present study compared to global‐scale studies that have found evident patterns of latitudinal changes in species richness (Jones & Cheung, 2015). Most of the AF grids are presumed to lose species, as already documented by Loyola et al. (2014), but even the higher rates of losses in the southeastern grids do not change the fact that, presumably, this will still be the richest region of anurans in the hotspot. A combination of potential mechanisms is argued to explain the highest AF anuran richness in the southeastern Brazil close to the Atlantic coast, such as the high humidity levels of the region (da Silva et al., 2012; Vasconcelos et al., 2010) and the rough topography that may had promoted higher rates of allopatric speciation in the region (Haddad & Prado, 2005).

On the other hand, although most CER grids are expected to lose species, most of the northeastern grids are expected to gain in species. These gains are a probable reflection of the expansion of common/widespread species since these grids have high beta diversity in the baseline climate, but the future climate change predictions evidence a homogenization of the anuran composition in this region (Figures 3 and 6; see complete discussion regarding beta diversity ahead). Although biodiversity losses are the main consequences regarding the human activity worldwide, biodiversity gains over some time period have also occurred in different regions of the world (Primack et al., 2018 and references therein). Our results highlight the probable occurrence of biodiversity gains in northeastern CER as a response to climate change, which therefore reinforces the value of considering this phenomenon to assist conservationists on effective practices to deal with higher rates of species replacement and maintenance of ecosystem processes and services (Primack et al., 2018). All in all, the climate change is not predicted to change the fact that the highest anuran richness in CER is found in the southern rim of the hotspot, but a considerable species loss is expected in the western and southwestern areas with current moderately‐to‐high richness. This region is in contact with the lowlands of the Pantanal floodplain, the largest contiguous extent of this habitat type on Earth that had been recently predicted to have no climatically suitable areas by 2050 for four generalist treefrogs as a consequence of the climate change effects (Vasconcelos & Nascimento, 2016).

Because different anuran species pools have been identified between and within the analyzed hotspots (e.g., Valdujo, Carnaval, & Graham, 2013; Vasconcelos, Rodríguez, & Hawkins, 2011; Vasconcelos et al., 2014), we expected that the main component structuring the patterns of beta diversity of both hotspots, irrespective of the climate scenarios, would be accounted for the species replacement along their geographic extents. These expectations were confirmed, but we also found different and interesting results for the climate change impacts on the anuran beta diversity in the AF and CER: (a) the anuran composition along the CER extension tends to be more homogeneous under the climate change scenarios, as indicated by the lowest beta diversity dissimilarity values across time in this hotspot, with an associated decrease in the importance of the turnover and nestedness values; (b) although the dissimilarity values of beta diversity in the AF are similar across time, which means that the degree of changes in the anuran composition along the AF is expected to be the same among the different time slices, the nestedness component has increased values in the climate change predictions while the turnover component decreases. The nestedness component becomes higher in the AF under the climate change probably as a reflection of the loss of climatically suitable areas for 37 species and the tendency for range contractions for AF anurans that resulted in species losses per cell (e.g., Lemes et al., 2014; Loyola et al., 2014; present study), which in turn have led more grid cells being subsets of other richer ones. Spatially speaking, there is no such a deep change in the distribution of beta diversity in the AF across time, so the use of beta diversity distribution patterns for conservation purposes in the AF (e.g., Legendre, 2014; Socolar et al., 2016) should not result in different strategies between current and future climate scenarios.

We found that a homogenization of the anuran fauna along the CER extent is presumed in future climate change scenarios compared to the baseline climate. Hence, the turnover and nestedness components decrease in importance for future predictions for the sake of the homogenization of the anuran fauna by 2050 and 2070. Although species loss is an important driver of the temporal change in species composition, it is their interaction with the arrival of new species that ultimately drives the beta diversity changes of communities across time (Molinos et al., 2015). Then, the higher rates of species gain in the CER compared to the AF, mainly located in the northeastern CER, might be responsible for the lower dissimilarity values of beta diversity in this hotspot by 2050 and 2070. Congruently, most of the grid cells with species gains under the climate change scenarios (Figure 3) are the same ones predicted to have high beta diversity in the baseline climate, but not anymore in the future climate change scenarios (see Figures 3 and 6), thus evidencing the range expansion of some species in this region. From a conservation point of view, different strategies might be considered in the CER from current to future actions considering the dynamic changes of the beta diversity distribution. Sites having unusual combination of species (i.e., grid cells with high beta diversity) are presumed to decrease in the northeastern region of the CER in future climate change scenarios, whereas a wider area of high beta diversity is expected by 2050 and 2070 in the southeast/southwest. Hence, a gradual decrease in conservation efforts in northern/northeastern areas of CER should be compensated by higher conservation efforts across time in the southeastern/southwestern region of the hotspot (e.g., Figure 6 may help decision‐makers to select specific and/or establish new conservation units, as well as establishing corridors between current and future predicted areas of high beta diversity; Socolar et al., 2016).

The potential impacts of climate change in the Brazilian hotspots are not expected to drastically change the distribution of the anuran richness gradients, but to negatively impact their whole extensions (i.e., cause species losses throughout the hotspots), except the northeastern CER region that is expected to gain in species. Areas having unique species composition are expected to decrease in northeastern CER, whereas an associated increase is expected in southeastern/southwestern areas under climate change. High beta diversity areas in future scenarios are expected to remain in the same locations as the prediction of the baseline climate in the AF, but the major tendency of species loss under climate change is expected to increase the nestedness component of the anuran beta diversity in the hotspot. Therefore, all these results suggest that the lack of similar climatically suitable area for most species will be the main challenge that they will face in the future. However, some concerns not addressed in our modeling framework may generate different patterns in the future: (a) anurans in the CER may have the opportunity to explore new savannah areas predicted to expand northwestward in projected climate change scenarios, whereas the AF anurans will have only few new forested areas projected in the southern rim of the hotspot (Nobre et al., 2016; Salazar, Nobre, & Oyama, 2007); (b) the richness gradients and beta diversity patterns might also be affected by the arrival of new species currently inhabiting the adjacent biomes (Loyola et al., 2013; Primack et al., 2018); and (c) the current rates of habitat loss that both hotspots have faced during the last century might cause even more species loss and the increase of the nestedness in the structure of the anuran beta diversity in the studied areas. Then, an integration of the present framework coupled to the above‐mentioned concerns is ideally the best procedure to obtain effective decisions for conservation actions that will therefore need to anticipate and accommodate such changes in climate (Luo et al., 2015; Molinos et al., 2015). Finally, gathering different biological metrics, as we did for anurans herein, and for a wide range of taxa and different future climate change scenarios, is an important step for conservation biogeography of the threatened biomes.

## CONFLICT OF INTEREST

None declared.

## AUTHOR CONTRIBUTIONS

TSV conceived the idea and designed the study; BTMdN and TSV collected and filtered the raw data; TSV and VHMP performed the statistical analyses and interpreted the results; TSV led the writing with substantial input from all authors.
